# Early stress exposure on zebrafish development: effects on survival, malformations and molecular alterations

**DOI:** 10.1007/s10695-024-01355-0

**Published:** 2024-05-14

**Authors:** David G. Valcarce, Alba Sellés-Egea, Marta F. Riesco, María-Gracia De Garnica, Beatriz Martínez-Fernández, María Paz Herráez, Vanesa Robles

**Affiliations:** 1 Cell Biology Area, Molecular Biology Department, Universidad de León, Campus de Vegazana S/N, 24071 León, Spain; 2 MicrosVeterinaria, Profesor Pedro Cármenes, Campus de Vegazana, 24007 León, Spain

**Keywords:** Zebrafish larvae, Stress, Malformations, Behaviour, Cartilage

## Abstract

The effects of stress during early vertebrate development can be especially harmful. Avoiding stressors in fish larvae is essential to ensure the health of adult fish and their reproductive performance and overall production. We examined the consequences of direct exposure to successive acute stressors during early development, including their effects on miR-29a and its targets, survival, hatching and malformation rates, larval behaviour and cartilage and eye development. Our aim was to shed light on the pleiotropic effects of early-induced stress in this vertebrate model species. Our results showed that direct exposure to successive acute stressors during early development significantly upregulated miR-29a and downregulated essential collagen transcripts *col2a1a*, *col6a2* and *col11a1a*, decreased survival and increased malformation rates (swim bladder, otoliths, cardiac oedema and ocular malformations), promoting higher rates of immobility in larvae. Our results revealed that stress in early stages can induce different eye tissular architecture and cranioencephalic cartilage development alterations. Our research contributes to the understanding of the impact of stressful conditions during the early stages of zebrafish development, serving as a valuable model for vertebrate research. This holds paramount significance in the fields of developmental biology and aquaculture and also highlights miR-29a as a potential molecular marker for assessing novel larval rearing programmes in teleost species.

**Supplementary Information:**

The online version contains supplementary material available at 10.1007/s10695-024-01355-0.

## Introduction

Stress has been defined as a systemic response to what an organism recognises as a threat (Yaribeygi et al. 2017). It is directly associated with changes in the physiology of the organism and even in its behaviour. Stress acts through various physiological mechanisms that affect the central nervous system (CNS) by dysregulating gene expression in the nervous system, ultimately altering hormone and neurotransmitter production (Yaribeygi et al. 2017). Stress can present two forms: acute or chronic (Thomson et al. 2020; Demin et al. 2021). Acute stress generates rapid alterations in neuronal activity, promoting a quick release of endocrine molecules and neurotransmitters to re-establish physiological homeostasis as quickly as possible (Demin et al. 2021). Chronic stress generates sustained changes in the organism driven by continued altered neurotransmitter and hormone production or gene expression modulation negatively affecting the organism, generating pathologies in multiple systems (Gauthier and Vijayan 2020). Consequently, stress can ultimately affect, in addition to behaviour (Ghisleni et al. 2012), metabolic processes (Heinkele et al. 2021), reproduction (Teng et al. 2020) or survival (Fan et al. 2019). Therefore, stress directly affects animal welfare. All the generated scientific knowledge in this topic has led to increased attention to animal welfare and its certification at animal production facilities by the scientific community, policy-makers and consumers; the aquaculture industry is directly affected by this growing interest (Campbell et al. 2021). In this context, the investigation of new biomarkers to assess animal stress (Aerts et al. 2015) is paramount. At aquaculture facilities, several stress sources can potentially cause pain and stress to individuals, such as feeding protocols, tank cleaning, population assessment and periodic control samplings (Braithwaite and Ebbesson 2014). The impact of stress on cultured species can occur during the larvae to adult life culture; the first stages of the life cycle are crucial to ensure the productive success of the batches and the fish welfare throughout their lives.

In the present work, we use zebrafish (*Danio rerio*), a popular teleost model species that has been used in a wide range of research areas, such as biomedical research (Choi et al. 2021; Aranda-martínez et al. 2022), ecotoxicology (Abe et al. 2021; Porto et al. 2023) or aquaculture-focused experiments (Dahm and Geisler 2006; Noble et al. 2017), among others. Zebrafish is an excellent model species due to its easy maintenance, prolificacy and external fertilisation (Swaminathan et al. 2023; Amar and Ramachandran 2023). The transparent zebrafish embryo is an exceptional model for studying the interplay between genetic and environmental influences. *Danio rerio* development is rapid, and embryos remain transparent throughout most of the embryogenesis phase, simplifying the monitoring of the progenies (Kimmel et al. 1995). Zebrafish shows a fast and precisely timed ontogeny, aiding the visual identification of developmental markers (Singleman and Holtzman 2014). Moreover, this model species presents a high genetic and physiological homology with other vertebrates, including humans (Amar and Ramachandran 2023; Lee et al. 2023). These are the many reasons why this species has been used to study the role of environmental influences and external stimuli on larval development (Rosa et al. 2022; Zhang et al. 2023), such as contaminants (Dai et al. 2014; Bhagat et al. 2020), drugs (Petersen et al. 2021; Rosa et al. 2022) or physiochemical alterations in the environment (Zhang et al. 2023).

In developing vertebrates, genetic factors must perfectly operate in the context of multiple cell interactions and environmental influences and variations. Therefore, regulation of gene expression plays a key molecular role in the development process and the modulation of the response to stressors. Among the different strategies involving the regulation of eukaryotic gene expression, in the present work, we focus on micro-RNAs (miRNA). These small non-coding RNA sequences are single-stranded RNAs between 18 and 26 nucleotides in length that act targeting specific messenger RNA (mRNA), consequently downregulating their gene expression by translational repression, mRNA cleavage and deadenylation (Ambros 2004). As an example of the crucial role of miRNAs in gene expression regulation in humans, these are responsible for regulating up to 60% of protein-coding genes (Hogg and Harries 2014).

Among the several miRNA families, in this work, we choose to focus on the miRNA-29 family (Horita et al. 2021), specifically on miR-29a. Previous results from our group have indicated a dysregulation on this non-coding RNA in zebrafish 7 days post-fertilisation (dpf) larvae derived from crossings involving chronically stressed males with control undisturbed females (Riesco et al. 2023). Like the vast majority of miRNAs, mature forms of the miRNA-29 family are highly conserved in different species, including humans, rats and zebrafish (Garcia-Concejo et al. 2018; Horita et al. 2021). miRNA-29a has been shown to play key roles in controlling early gene expression (Nanda et al. 2020), regulating extracellular matrix (Chuan-hao et al. 2016) and mineralisation (Horita et al. 2021), among others (Shi et al. 2020; Horita et al. 2021). Therefore, this miRNA affects relevant biological processes of great importance in the field of aquaculture since the existence of malformations or alterations in early development can negatively affect companies in this industry economically (Eissa et al. 2021).

We hypothesise that short periods of stress suffered during early larval stages in zebrafish can alter miR-29a levels, producing phenotypic alterations in larvae. The aim of the present study is to generate knowledge about potential stress biomarkers in teleost larvae. These biotechnological tools will be valuable for the aquaculture industry, allowing the evaluation of potential new larvae culture protocols by determining stress levels not only from survival, malformation rates or biometry parameters but also from a molecular prism.

## Methods

### Fish husbandry and larvae exposure to stressors

**Fig. 1:**
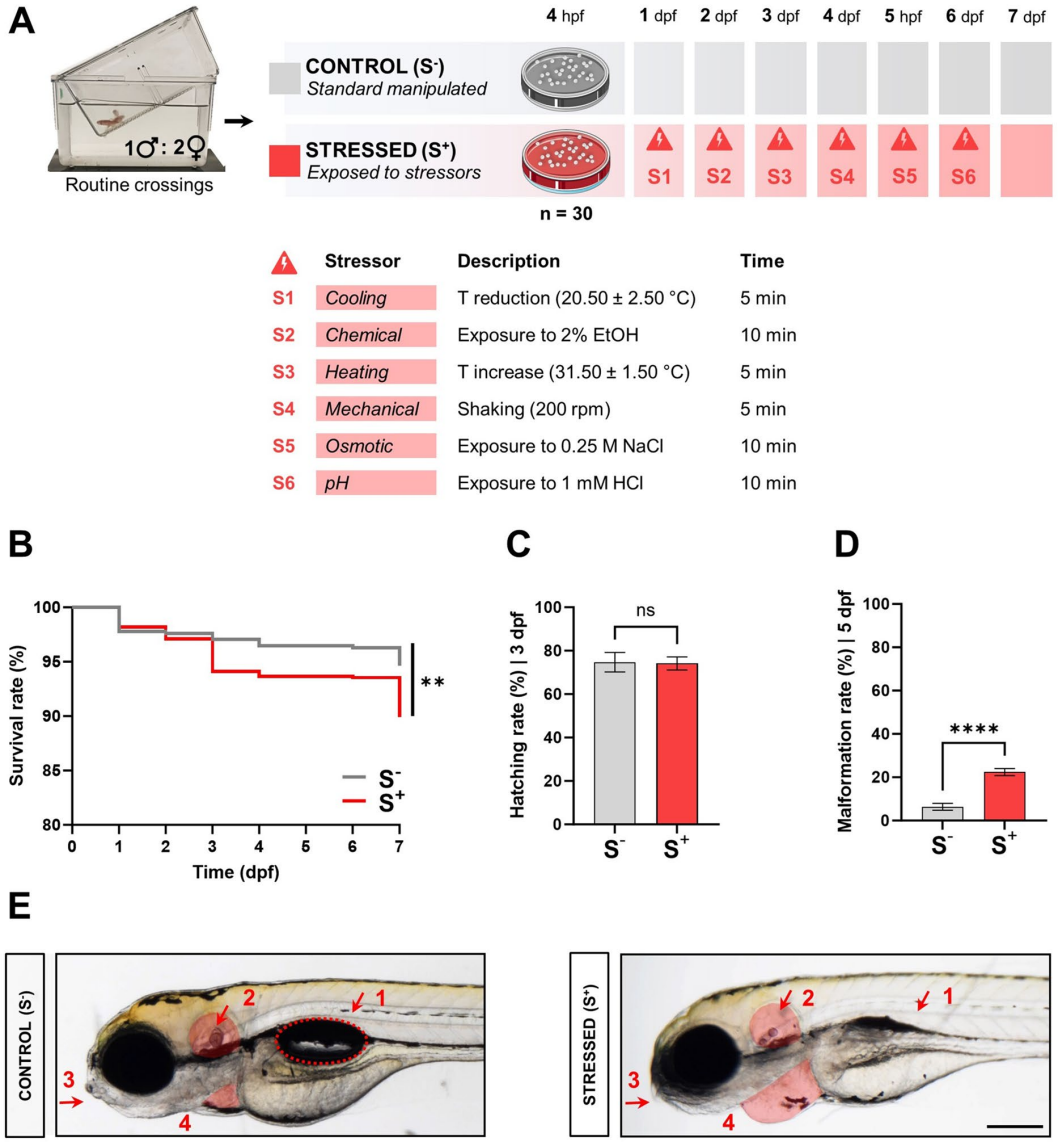
Global impact of exposure to successive acute stressors on zebrafish development.** A** Experimental design. Biological replicates (30 viable and normally developed 3-hpf embryos/plate at the beginning of the experiment) obtained from routine crossings (1♂:2♀). Two experimental conditions: control (S^−^, in grey), cultured larvae undisturbed during the trial, and stressed (S^+^, in red), stressed larvae. S^+^ group larvae exposed to six different stressors, labelled as S1 to S6 (one per day from day 1 to day 6 in the trial). Each acute stressor, day of culture at which this was applied and time of exposure are described. **B** Percentage of larvae survival (Kaplan–Meier curves; Mantel–Cox method (S^−^, *N* = 18; S^+^, *N* = 31; *n*_initial_ = 30 embryos / biological replicate) during the experiment. **C** Hatching rate (%) at 72 hpf (S^−^, *N* = 18; S^+^, *N* = 31; *n*_initial_ = 30). **D** Malformation rate (%) at 5 dpf (S^−^, *N* = 18; S^+^, *N* = 31; *n*_initial_ = 30 embryos / biological replicate). **E** Phenotypes reported at 5 dpf in both experimental groups. Malformed individuals from the stressed (S^+^) group show uninflated swim bladder (1), altered otoliths development (2, in red), shorter jaw (3) and lymphoedema formation (4, in red). Data are presented as mean ± SEM. *p < 0.0500; **p < 0.0100; ****p < 0.0001; ns, no statistically significant differences (*p* > 0.0500)

Zebrafish eggs (*Danio rerio;* AB wild-type strain) were obtained from natural spawning family crossings at the Animal Research and Welfare Service facilities of the University of Léon (Spain). Fertilised eggs were selected within the blastula period at 3 h post-fertilisation (hpf) and incubated in embryo medium (EM: 0.137 M NaCl; 5.4 mM KCl; 0.25 mM Na_2_H PO_4_; 0.44 mM KH_2_ PO_4_; 1.3 mM CaCl_2_; 1.0 mM Mg SO_4_: 4.2 mM NaH CO_3_) at 27.5 ± 0.5 °C during the entire experiment. The biological replicate was defined as a Petri dish (⌀: 5.5 cm) filled with 8 mL of EM containing 30 individuals. The daily upkeep included an EM volume change every 24 h. Eight different crossings were used to produce the biological replicates to work with different genetic backgrounds. Two culture conditions (control: S^−^; stress: S^+^) were used during the first 7 days of life of the individuals. Stress induction consisted in a 6-day protocol including different types of acute stressors. Six stressors (Yeh et al. 2013; Bai et al. 2016) were used in this protocol (one acute stressor per day from 1 to 6 dpf; Fig. 1A): (1) cooling, decreasing the temperature in the Petri dish to 20.50 °C ± 2.50 °C for 5 min at 1 dpf; (2) chemical, 2% EtOH exposure for 10 min at 2 dpf; (3) heating, raising the temperature in the Petri dish to 31.50 °C ± 1.5 °C for 5 min at 3 dpf; (4) mechanical, shaking (orbital shaker; 200 rpm) for 5 min at 4 dpf; (5) “osmotic”, 0.25 M NaCl exposure for 10 min at 5 dpf; and (6) “pH”, 1 mM HCl exposure for 10 min at 6 dpf. All the stressors were applied outside the incubator. While stressors were being applied, the S^−^ replicates were also removed from the incubator to avoid potential bias in the resulting data.

### Physiological response to stressors

#### Survival, hatching and malformation percentages

Mortality was assessed by daily (1 to 7 dpf) removal of dead individuals from the dishes. The hatching rate (percentage of hatched larvae per live animals within the biological replicate) was calculated at 3 dpf. Malformation rate (percentage of malformed larvae per live animals within the biological replicate) was calculated at 5 dpf under stereomicroscope (SMZ1500, Nikon, Tokyo, Japan). The malformations evaluated in this study included: uninflated swimming bladder, non-canonical otoliths, jaw malformations and lymphoedema formation. Representative examples of control and malformed larvae were anaesthetised in 0.02% tricaine (MS222, Sigma-Aldrich, MO, USA) at 5 and 7 dpf for image and video collection from a lateral perspective using a Nikon DS-Fi3 digital camera.

#### Cranial and eye development

Larvae cartilage development analysis was performed using whole-mount alcian blue staining in 7-dpf specimens. Larvae from both groups showing normal and malformed phenotypes were fixed in 4% (w/v) phosphate-buffered saline (PBS)-buffered paraformaldehyde (4 °C, o/n). After fixation, samples were stored at 4 °C in 70% EtOH. Alcian blue staining was performed according to Valcarce et al. (2017) (Valcarce et al. 2017). Stained larvae were ventrally placed under a stereomicroscope and photographed. The resulting images were processed with ImageJ software (Schneider et al. 2012) to obtain two measurements of the eye dimensions, labelled as M1 and M2, respectively, corresponding to the major and minor axes of the virtual ellipse generated from the eye area (Fig. 4B). Images were also used to measure head length, ceratohyal cartilage length, lower jaw length and Meckel’s–palatoquadrate angle (M-PQ; Fig. 5A).

### Larval behaviour

For the behavioural assessment, larvae swimming activity was first measured at 7 dpf by counting the number of immotile larvae in each plate after 1 min. Then, we performed a previously described novel tank test (NTT) for zebrafish larvae (Valcarce et al. 2017). Live larvae showing a motile activity in their housing dish (8 animals per biological replicate; replicate size: S^−^: *n* = 18; S^+^: *n* = 21) were randomly selected and evaluated. Each larva was individually placed in a novel Petri dish (⌀: 5.5 cm) and let to adapt to the new environment for 1 min; subsequently, animal behaviour was video recorded for 5 min. Tracker software (physlets.org/tracker/) was used for larvae swimming tracking. Each resulting trajectory was processed using a virtual 16-zone grid pattern (8 inner and 8 outer subareas) to compare fish exploration (Fig. 3B).

### RNA extraction

Total RNA was isolated from 25 randomly selected 7-dpf larvae per replicate (9 biological replicates per experimental group) using the miRNeasy Tissue/Cells Advanced Mini Kit (Qiagen, Hilden, Germany) and following the manufacturer’s protocol. Briefly, to ensure complete tissue lysis and RNase inactivation, 260 µL of Buffer RLT was added to the samples, and afterwards, they were disrupted and homogenised. After centrifugation and supernatant recovery, 80 µL of Buffer AL was added to each sample to optimise gDNA removal in the gDNA eliminator spin column. Subsequently, 20 µL of Buffer RPP was added to the flow-through to precipitate inhibitors by centrifugation. Isopropanol was then added to the supernatant containing RNA, and the sample was applied to the RNeasy Mini spin column, where total RNA bounds to the membrane. Finally, RNA, including miRNA and other small RNA, was eluted in 30 µL of RNase-free water. Purity and quantity of the resulting RNA samples were analysed using a DeNovix® spectrophotometer (DeNovix Inc., Wilmington, NC, USA), while RNA integrity was assessed on agarose gel.

### Reverse transcription

Total RNA (starting from 10 ng) was reverse transcribed using the TaqMan MicroRNA Reverse Transcription Kit (Applied Biosystems by Thermo Fisher Scientific, Vilnius, Lithuania) with specific TaqMan Small RNA probes (5 ×) for dre-miR-29a (MI0001938) and dre-miR-92a (MIMAT0001808) following the manufacturer’s guidelines. miR-92a-3p was selected as reference miRNA as previously reported in zebrafish larvae samples (Riesco et al. 2019). For mRNA reverse transcription, complementary DNA (cDNA) was obtained from 2 μg total RNA using the High-Capacity RNA-to-cDNA Kit (Applied Biosystems by Thermo Fisher Scientific, Vilnius, Lithuania), following the manufacturer’s protocol.

### Quantitative polymerase chain reaction

All quantitative polymerase chain reaction (qPCR) experiments were conducted in technical triplicates (*n* = 9) on a QuantStudio™ 1 Real-Time PCR system (Applied Biosystems by Thermo Fisher Scientific, MA, USA) under standard thermal conditions. The reactions for the quantification of miRNA expression levels were prepared following the protocol for TaqMan Universal PCR master mix II (Applied Biosystems by Thermo Fisher Scientific, Vilnius, Lithuania). The reactions for the quantification of mRNA expression levels contained 10 μL of SYBR Green PCR Master Mix (Applied Biosystems by Thermo Fisher Scientific, Warrington, UK), 1 μL of each 10 μM forward and reverse primer, 2 μL of cDNA sample and 6 μL of molecular biology degree water up to 20 μL. We analysed gene expression changes of transcripts related to retinoic acid (*rarga*, *rxrba*, *rxrbb*) and cartilage (*col2a1a*, *col2a1b*, *col6a2*, *col6a3*, *col11a1*). A list of primers can be found in Additional file 1: Table S1. We performed a melting curve analysis to determine the specificity of qPCR reactions. The BestKeeper tool (Pfaffl et al. 2004) was used for the selection of the optimal housekeeping gene (HKG) in the experiment. Three reference genes were studied: actin beta 2 (*actb2;* (Riesco et al. 2014)), eukaryotic translation elongation factor 1 alpha 1a (*eef1a1a*; (Tang et al. 2007)) and ribosomal protein S18 (*rps18;* (Divisato et al. 2016)). Gene expression was normalised relative to *actb2* as HKG, since this was reported to be the best candidate exhibiting the lowest variation and, therefore, the most stably expressed of the three studied HKGs.

### Histology

At 7 dpf, control and malformed larvae were fixed in 4% (w/v) PBS-buffered paraformaldehyde (4 °C, o/n). Then, samples were pre-embedded in 1% agarose (iNtRON Biotechnology, Seongnam, Korea) to facilitate the correct orientation during sectioning. For microtome sectioning, all fish were dehydrated and paraffin embedded in a Myr STP-120 tissue processor. Serial orthogonal and sagittal 2.5-μm-thick sections were obtained using a Leica RM2255 rotary microtome. Sections were stained with haematoxylin–eosin (H&E) and Masson’s trichrome to visualise mesenchymal tissue. Sections were dehydrated and mounted with DPX mounting medium (Sigma-Aldrich,MO, USA). We observed the resulting sections under a light microscope Olympus BX61 and took microphotographs using a DS-Fi3 (Nikon, Tokyo, Japan) camera attached to a E600 (Nikon, Tokyo, Japan) microscope. Morphometric analysis was performed using ImageJ software (v1.54 g; (Schneider et al. 2012)).

### Statistics and reproducibility

All statistical analyses were conducted using GraphPad Prism 9.0 (GraphPad Software, CA, USA). All assays were performed on at least 9 replicates (*N*), and each consisted in a sample size (*n*) of 9–30 fish. The comparison of survival curves was performed with a log-rank (Mantel–Cox) test. Data from the remaining experiments were tested for deviation from the Gaussian ideal using the Shapiro–Wilk normality test. For normally distributed data, experimental groups were compared using a two-tailed *t* test (with Welch’s correction when the variances of the variables were not equal after running a *F* test). For non-parametric data, a Mann–Whitney test was run. All data are shown as mean ± SEM (*p < 0.0500; **p < 0.0100; ****p < 0.0001; ns, no statistically significant differences).

## Results

### Physiological impact of daily stressors on zebrafish development

To confirm the impact of exposure to successive acute stressors during early stages on the development of the organism, embryos were monitored from 24 hpf to 7 dpf. The Kaplan–Meier survival curve comparison shows statistically significant differences (*p* = 0.0018) between control larvae and those exposed to stressors (Fig. 1B). However, the survival rate was high in both groups, over 90% in both cases, reporting a subtle effect of the selected combination of stressors on larvae viability.

No statistically significant differences (*p* = 0.7854) were found between the two groups in the hatching percentage at 72 hpf (Fig. 1C). Collected data showed almost equal mean values for this parameter in both experimental groups (around 75%), revealing an absence of impact of the exposure to successive acute stressors on embryo hatching ability. However, statistically significant differences (*p* < 0.0001) were found for the malformation ratio at day 5. The combination of acute stressors increased by 3.7 times the appearance of altered phenotypes at this key point in time in the S^+^ biological replicates compared with that in the S^−^ standard cultured ones (S^+^, 6.411 ± 1.585%; S^−^, 22.42 ± 1.614%; Fig. 1D). Malformed larvae in the S^+^ group presented a combination of the following features: (1) uninflated swimming bladder, (2) non-canonical otoliths, (3) jaw malformations and (4) lymphoedema formation (abundant cases of pericardial lymphoedema; Fig. 1E). These alterations were also detected at the end of the trial (7 dpf), and in some cases were even more noticeable at this key point (Additional file 2: Video S1).

### Molecular impact of daily stressors on larvae miR-29a levels

**Fig. 2:**
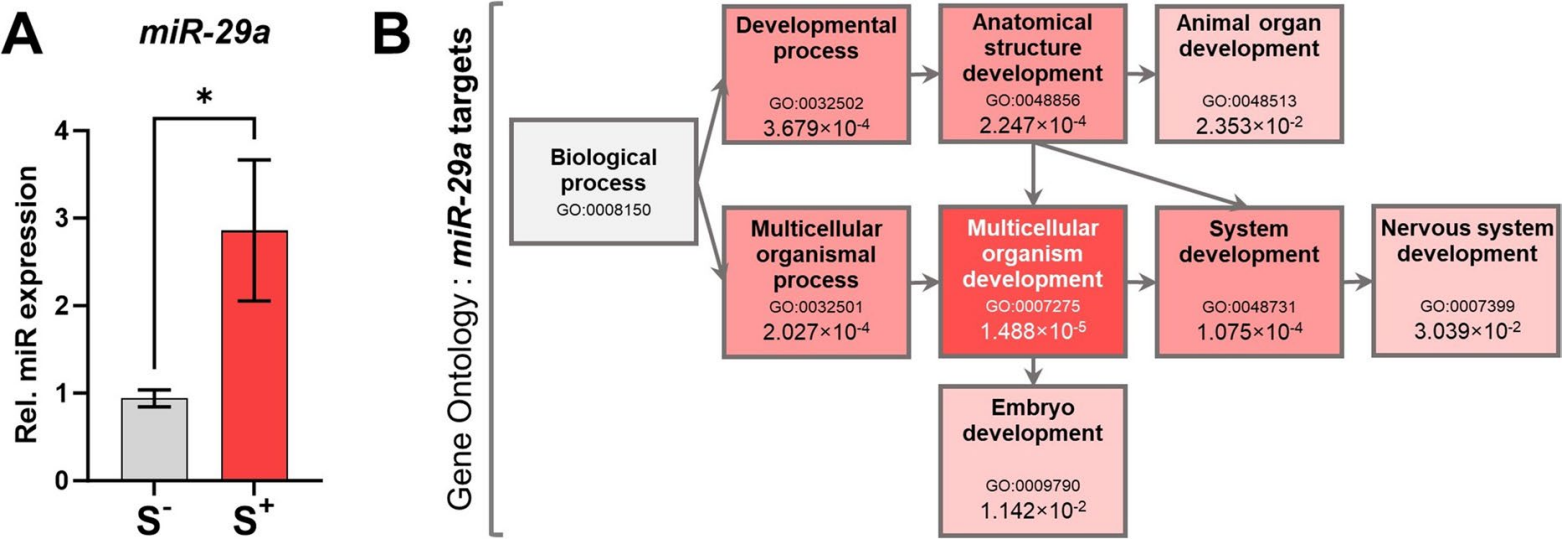
miR-29a analysis. **A** Relative miR-29a expression (*N* = 9; *n* = 25).** B** Gene ontology results (g:Profiler; biological processes) for the 2208 miR-29a targeted genes (predicted by TargetScanFish). Significantly enriched terms are displayed with a reddish shadow corresponding to their enrichment *p*-values. Grey-shadowed item corresponds to the root of the domain. Term ID, description and enrichment *p*-value are shown for each term. Data in A are presented as mean ± SEM. *p < 0.0500

We studied the expression of zebrafish miR-29a as this has been explored as a regulatory molecule involved in stress response modulation both in animal models (Riesco et al. 2023) and humans (Maffioletti et al. 2021). Larvae from the S^+^ replicates showed a statistically significant miR-29a upregulation (*p* = 0.0129) compared with their non-disturbed control counterparts. The qPCR analysis reported a threefold change increase in the expression of miR-29a (Fig. 2A).

Considering TargetScanFish data, miR-29a potentially targets a list of 2208 mRNAs. The complete list of targeted genes can be found in Additional file 3: Table S2. Gene ontology (GO) enrichment analysis is presented in Fig. 2B where statistically significant GO entries (multicellular organism development (GO:0007275; *p* = 1.488 × 10^−5^), system development (GO:0048731; *p* = 1.142 × 10^−2^), multicellular organismal process (GO:0032501; *p* = 2.027 × 10^−4^), anatomical structure development (GO:0048856; *p* = 2.247 × 10^−4^), developmental process (GO:0032502; *p* = 3.679 × 10^−4^), embryo development (GO:0009790; *p* = 1.142 × 10^−2^), animal organ development (GO:0048513; *p* = 2.353 × 10^−2^) and nervous system development (GO:0007399; *p* = 3.039 × 10^−2^)) are displayed in a tree connecting edges between the term nodes for a clearer description of the biological context.

### Early stress exposure impact on zebrafish behaviour

**Fig. 3:**
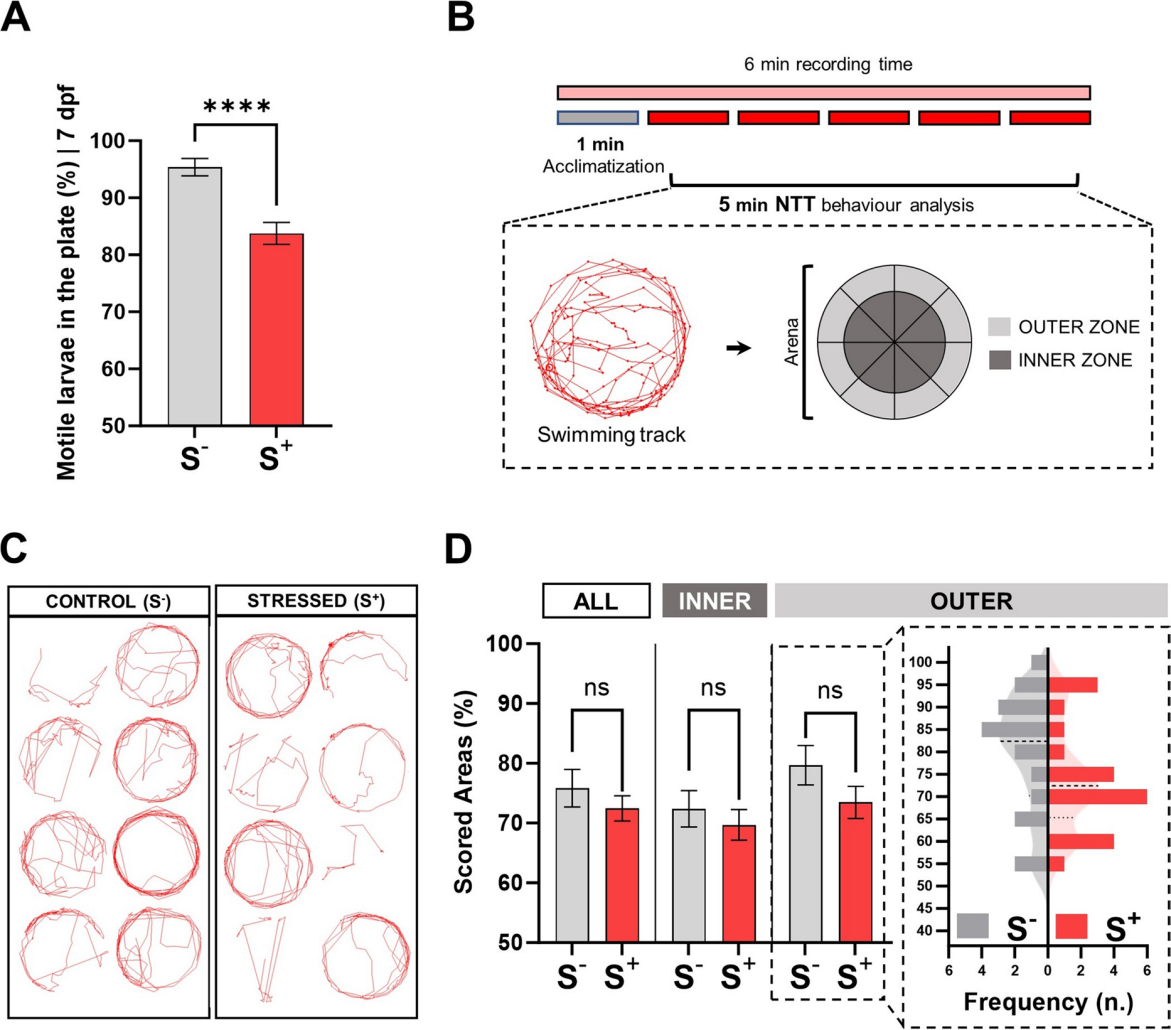
Larvae locomotor activity analysis. **A** Percentage of motile larvae per plate at 7 dpf (S^−^, *N* = 18; S^+^, *N* = 31; *n*_initial_ = 30 embryos / biological replicate). **B** Diagram representing the novel tank test (NTT) used in the experiment. Examples of tracks reported by Tracker software (red line). Virtual grid (1 arena; 16 zones: 8 inner (dark grey) and 8 outer zones (light grey)) used for movement quantification in terms of zone exploration. **C** Representative examples of individual swimming tracks in both experimental groups. **D** Registered areas in both groups studying the total zones (ALL), central zones (INNER) and perimetric zones (OUTER) (S^−^, *N* = 18; S^+^, *N* = 21; *n* = 8 tracks / biological replicate). Histogram shows data corresponding to the outer sub-analysis. Control (S^−^, in grey), cultured larvae undisturbed during the trial; stressed (S^+^, in red), stressed larvae. Data are presented as mean ± SEM. ****p < 0.0001; ns, no statistically significant differences (*p* > 0.0500)

Exposure to stressors caused higher rates of immobility in the S^+^ experimental group (*p* < 0.0001). While in the control group the number of immobile larvae on the plate at day 7 dpf represented around 5% of the total, the replicates subjected to stress during their early stages of development showed a mean value of approximately 15% of the individuals (Fig. 3A). The analysis of larvae behaviour using the chosen NTT (Fig. 3B) allowed to evaluate the exploration of pattern preference in a new environment of the motile larvae from each experimental group. Resulting tracks (Fig. 3C) were processed focusing on the whole new area (all), the peripheral zone (outer) and the central zone of the explorable arena (inner). No statistically significant differences were found either in the global arena (*p* = 0.3663), the outer (*p* = 0.5014) or the inner (*p* = 0.1472) scoring comparison (Fig. 3D). Thus, motile larvae from each experimental group showed similar behaviour patterns, with a general scoring of around the 70% of the new exploration arena in both groups (Fig. 3C). Specimens from both groups tended to explore more the peripheral zone (outer) of the new arena, a behaviour usually found in experiments evaluating swimming patterns in zebrafish larvae (Schnörr et al. 2012). Although no statistically significant differences were found between experimental conditions, we deemed worthy to highlight the distribution of the two groups in the outer sub-evaluation (Fig. 3D, dotted-line box).

### Effects of early stress exposure on eye development

**Fig. 4:**
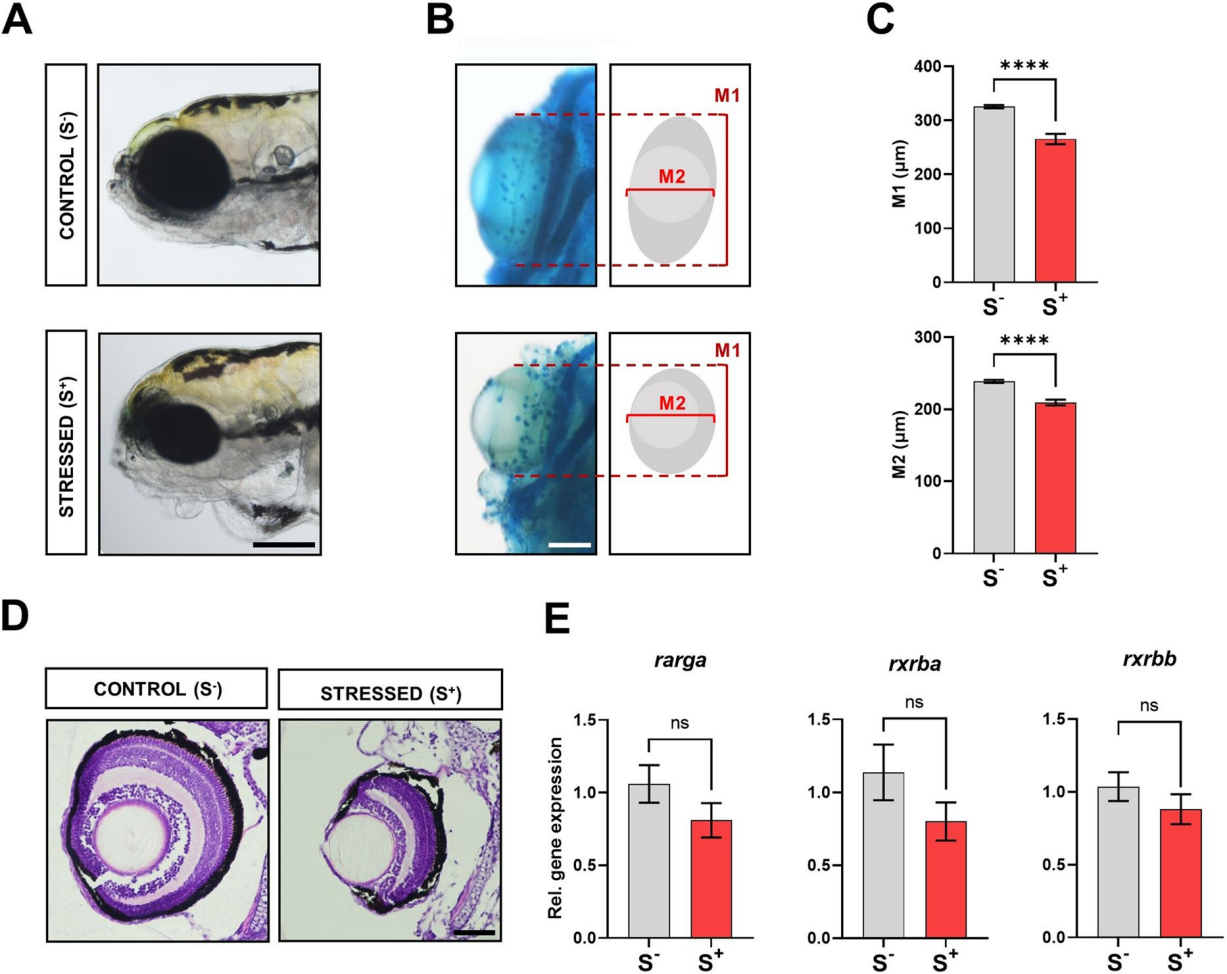
Impact of exposure to successive acute stressors on zebrafish eye development.** A** Phenotypes reported at 7 dpf in both experimental groups showing a control individual and a malformed one. Note the reduction in eye size. Scale bar: 250 μm.** B** Detail of ventrally disposed alcian blue-stained individuals focussing on the eyes. Eye dimensions were quantified using two measurements: M1 and M2, respectively, corresponding to the major and minor axes of the virtual ellipse generated from the eye area. Scale bar: 100 μm. **C** Comparison of the two established measurements (M1 and M2) for eye size analysis (S^−^, *N* = 12; S^+^, *N* = 16). **D** Dorsal H&E histological (1.5 μm thick) analysis of zebrafish eyes in control and malformed individuals from both experimental groups. Scale bar: 50 μm. **E** Relative gene expression of three retinoic acid-related genes (miR-29a targets) in 7-dpf larvae from both experimental groups (*N* = 9; *n* = 25 larvae / biological replicate): *rarga*, *rxrba* and *rxrbb*. Control (S^−^, in grey), cultured larvae undisturbed during the trial; stressed (S^+^, in red), stressed larvae. Data are presented as mean ± SEM. ****p < 0.0001; ns, no statistically significant differences (*p* > 0.0500)

To further explore the effects of early stress on larvae, we analysed eye development. At 7 dpf, one of the most evident subjective features (Fig. 4A) of the malformed phenotype was microphthalmia (abnormally small eyes). We corroborated this subjective observation by measuring eye size in alcian blue-stained larvae (Fig. 4B). The quantification of the size of both the major (M1) and the minor (M2) axes of the virtual ellipse corresponding to the area occupied by the eyes revealed statistically significant differences (*p* < 0.0001; Fig. 4C). The histological study of the eye corroborated a different tissular architecture when normally developed and malformed specimens were compared (Fig. 4D). Finally, we performed gene expression analyses on three retinoic acid (RA)-related genes: RA receptor gamma a (*rarga*), retinoid × receptor beta a (*rxrba*) and retinoid × receptor beta b (*rxrbb*). We focused on RA genes because RA acts as a crucial signal for development of the vertebrate eye by governing the transcriptional regulatory activity of RA receptors (RARs) (Duester 2008); also, the selected three transcripts are targeted by miR-29a (Additional file 4: Figure S1A). Gene expression results did not reveal statistically significant differences between groups (*p* > 0.0500); however, a downregulation trend was found for the three studied genes in the stress-exposed experimental group (Fig. 4E).

### Effects of early stress exposure on cartilage development

**Fig. 5:**
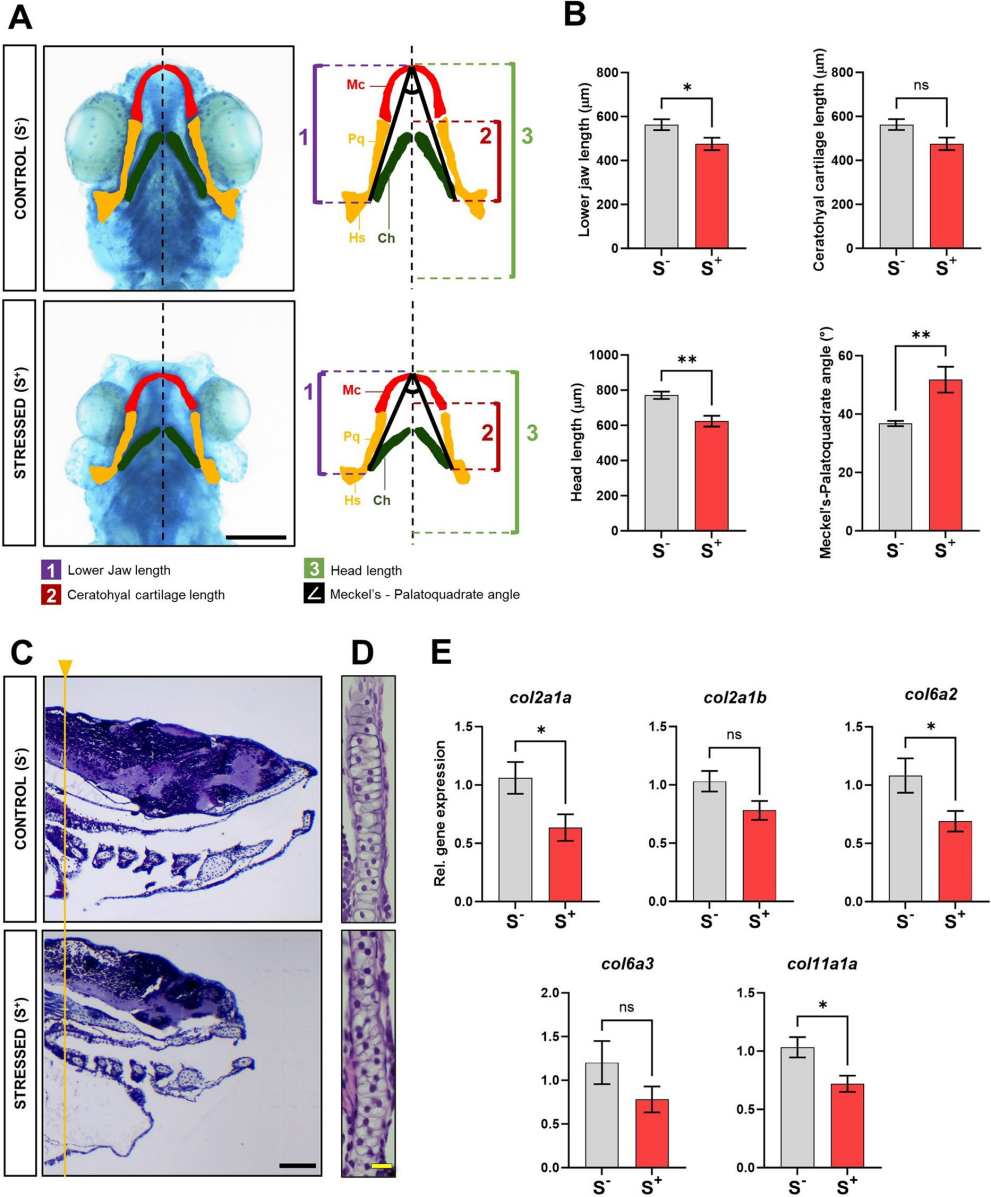
Impact of exposure to successive acute stressors on zebrafish cartilage development. **A** Representative images of alcian blue-stained 7-dpf control and malformed larvae (ventrally disposed). Scale bar: 200 μm. Right placed diagrams highlight specific cranioencephalic cartilages in different colours for easier interpretation: Meckel’s cartilage (Mc, red), palatoquadrate-hyosymplectic (Pq Hs, yellow) and ceratohyal (Ch, dark green). Four measures were quantified with ImageJ: (1) lower jaw length, (2) ceratohyal cartilage length, (3) head length and (∠) Meckel’s–palatoquadrate (M-PQ) angle. **B** Comparisons for lower jaw length (µm), ceratohyal cartilage length (µm), head length (µm) and M-PQ angle (°) (S^−^, *N* = 10; S^+^, *N* = 11). **C** Lateral Masson’s trichrome stained histological analysis (1.5 μm thick) of zebrafish cranioencephalic area in control and malformed individuals from both experimental groups. The yellow line represents a reference to display the photographs according to the position of the ceratobranchial cartilage 5. Scale bar: 50 μm. **D** Magnified views of trabecula chondrocytes (H&E staining). Scale bar: 10 μm. **E** Relative gene expression of five collagen genes (miR-29a targets) in 7-dpf larvae from both experimental groups (*N* = 9; *n* = 25 larvae / biological replicate): *col2a1a*, *col2a1b*, *col6a2*, *col6a3* and *col11a1a*. Control (S^−^, in grey), cultured larvae undisturbed during the trial; stressed (S^+^, in red), stressed larvae. Data are presented as mean ± SEM. *p < 0.0500; **p < 0.0100; ns, no statistically significant differences (*p* > 0.0500)

We decided to further study collagen-related parameters due to the abnormal otoliths, the reduction in the size of the jaw (micrognathia) and the evident alteration of the cranioencephalic development that we found in the malformed specimens of the S^+^ group (Fig. 1A) and also because miRNA-29a had already been shown to play key roles in regulating extracellular matrix (Chuan-hao et al. 2016) and mineralisation (Horita et al. 2021). First, we performed specific alcian blue staining for cartilage (main structural tissue at this life stage in the developing skeleton), tissue characterised by a collagen-rich extracellular matrix. The whole mount staining revealed clear severe anomalies in the cartilages (Fig. 5A). Qualitatively, malformed larvae showed shorter heads and shorter cartilages. The detailed image analysis with ImageJ (Fig. 5A; diagram) reported statistically significant differences in three of the measurements (Fig. 5B). Lower jaw length and head length were reported to be statistically significant shorter in the malformed larvae (*p* = 0.0323 and *p* = 0.0010, respectively). Similarly, the M-PQ angle was found to be wider (*p* = 0.0070) in malformed larvae (51.80 ± 4.448) compared with that in the control ones (36.75 ± 0.8961). The ceratohyal cartilage length did not reveal statistically significant differences (*p* = 0.1698). We further analysed histologically both the malformed and the canonical larvae (Fig. 5C). The reduction of the head length and abnormal shorted development of cranioencephalic zebrafish cartilages was corroborated in the histological sections. The detailed study of the trabecula chondrocytes in the malformed 7-dpf larvae (Fig. 5D) showed constricted altered chondrocytes, alteration of the characteristic stack-of-coins shape and lower cell volume, presumably affecting the overall craniofacial morphology. To confirm these tissular observations, we performed qPCR analyses for five collagen genes: *col2a1a*, *col2a1b*, *col6a2*, *col6a3* and *col11a1*, all of them targeted by miR-29a (Additional file 4: Figure S1B). Gene expression results revealed a global downregulation trend for the five selected genes (Fig. 5E). In line with the phenotypic and histological results, three of the collagen genes showed statistically significant differences: *col2a1a* (*p* = 0.0286), *col6a2* (*p* = 0.0365) and *col11a1a* (*p* = 0.0128).

## Discussion

In aquaculture, stress is a major concern, as the presence of negative stimuli in hatcheries can predispose fish to growth and health problems and eventually lead to malformations, diseases and increased mortality rates (López-Olmeda et al. 2012; Sánchez-Vázquez et al. 2019; Abdel-Tawwab et al. 2019). Stressors can alter the internal balance of the organism, generating different biochemical, physiological and even behavioural responses to return to the natural homeostasis (Canzian et al. 2021). Beyond the impact that stress can cause in industrial animal production, currently, the scientific community and producers must ensure that animals live in optimal welfare conditions in the facilities (European Commission 2021). Our group has focused lately on the evaluation of the effects of chronic stress on teleost spermatogenesis and its impact on the resulting progenies derived from chronically stressed males (Valcarce et al. 2023a) and in deepening the knowledge on how different protocols reducing the impact of anthropogenic sources of stress can improve fish welfare (Valcarce et al. 2023b). In the present work, we aim to gain insight into a potential stress biomarker in teleost larvae, i.e. microRNA-29a. We focus on this regulation molecule because previous ribonucleic acid sequencing (RNAseq) data from our lab has shown a statistically significant modulation in this miRNA in 7-dpf larvae whose male progenitors had been exposed to chronic stress covering more than one spermatogenic wave in the zebrafish model (Riesco et al. 2023). Herein, we evaluate whether a direct exposure to a battery of successive acute stressors during the first stages of development (1–6 dpf) also modulates the expression of this miRNA. Stress exposure in early stages may be especially relevant, since this represents a crucial phase in ensuring a future successful fish culture. Avoiding stress sources in larvae can be beneficial in ensuring the health of adult fish and adequate reproductive performance in future breeders. Larvae may be exposed to several possible stress sources and to important physiological changes (Pederzoli and Mola 2016), hence the importance of studying stress consequences in these stages. To induce stress in larvae, we selected a set of acute stressors previously published to rise cortisol levels in zebrafish larvae ((Yeh et al. 2013; Bai et al. 2016); Fig. 1A). Zebrafish larvae are excellent teleost models for testing external stimuli given their characteristics (Horzmann and Freeman 2018; Busse et al. 2020) and the ease to perform this kind of trials by manipulating the embryo’s incubation media. The chosen temporal frame to apply the acute stressors (1–6 dpf) was established as the range of time during which the animal cannot feed autonomously, and therefore, survival of larvae is mainly affected by environmental conditions, their genetic background and the quality of the reserves of their yolk sac. As expected, exposure to stressors during this sensitive developmental period affected key parameters in the progenies. Although not drastically, survival curve comparison was revealed to be different between groups (Fig. 1B). These results are logical, since the stressors used in this work were only induced for very short periods of time (5–10 min) and were not lethal for most of the specimens. The absence of high mortality rates can be advantageous in highlighting molecular and phenotypic alterations that might otherwise be masked by lethality. These rates also validate our experimental design, allowing for the comparison of our approach in the laboratory with the conditions in aquaculture facilities, where individuals are theoretically protected from constant aggressive stressors, such as heavy metals that cause high mortality rates by being present either as a pulse or in a constant manner in the environment (Chen et al. 2019). We also evaluated the hatching rate at 72 hpf and the malformation rate at 5 dpf, the moment in which major morphogenesis are completed in *Danio rerio* (Chu and Sadler 2009). Studying the hatching rate is crucial in aquaculture-focused studies since shorter hatching times mean cost savings and higher efficacy for fish farmers, and hatching success is a key indicator of correct development in an artificial environment (Watson and Chapman 1996). In the present work, hatching rate was not affected by the stress protocol (Fig. 1C). Note that, at 72 hpf, the survival curve of stressed individuals drops compared with that of the controls. This pattern in the survival curves could contribute to the results obtained for the hatching rate. Contrary to hatching rate results, the malformation rate showed a statistically significant difference between routinely cultured and stressed larvae (Fig. 1D). In teleost, malformations appear early in development, and these are frequent in reared larvae. The malformation rate is generally assumed in the aquaculture facilities as an indicator reflecting culture conditions (Chandra et al. 2024). The importance of the malformation rate in the industry is based on the driven decreased growth rate, increased mortality, increased production costs and eventually decreased market price. The stress induction in our experiment led to a malformation rate around 20% (Fig. 1D). The malformed 5-dpf larvae presented a combination of deformities, including a pericardial oedema, uninflated swim bladder, incorrect jaw elongation, inappropriate yolk sac resorption and non-canonical otolith development (Fig. 1E). These are typical malformations found in zebrafish under jeopardising culture conditions (Xu et al. 2022; Zhang et al. 2023; Yuan et al. 2023). Since our main goal in this work is to integrate the observation of phenotypic alterations derived from stress with potential deregulations of regulatory molecules, we studied the expression of miR-29a in the larvae at the end of the experiment. Besides our previous studies indicating a link between stress and this miRNA (Riesco et al. 2023), other groups have previously suggested a relation between miR-29a expression pattern and stressful conditions. In humans, this miR has been proposed as a neuroendocrine stress response marker (Liang et al. 2018) showing overexpression in type 2 diabetes mellitus patients. Also, an external stressful stimulus (chronic academic stress) promoted its overexpression (Honda et al. 2013). Cell culture-focused studies have also related miR-29a overexpression with endoplasmic reticulum (ER) stress-induced apoptosis (Nolan et al. 2016). Moreover, accumulating evidence correlates the regulatory role of miR-29a in different cancer types (Wu et al. 2019; Huang et al. 2022) and other pathologies, such as pulmonary fibrosis, hepatic fibrosis and aneurysm by targeting extracellular matrix molecules (Matsumoto et al. 2016; Smyth et al. 2022). miR-29a overexpression registered here (Fig. 2A) is in accordance to these previous results linking this non-coding RNA with a stressful environment for the organism. Thus, the higher ratio of malformations registered at 5 dpf in the stressed group (S^+^) may be partially explained by the overexpression of this miRNA, since the gene ontology exploration of its targeted mRNAs (Fig. 2B) globally suggested biological process entries related to development (embryo, anatomical, organ, nervous system, systems). Considering the phenotypes registered in the malformed larvae in the stressed group, we focused on three features: behaviour, reduction of eye size (microphthalmia) and reduction of jaw elongation (micrognathia). These foci of attention were also selected because these are crucial also from the aquaculture industry perspective. Aberrant behaviours are clear indicators of non-optimal husbandry conditions (Ingebretson and Masino 2013). Although in the present study it was not studied in detail whether the individuals in the stressed group are blind, based on the phenotype found, it is reasonable to think that the vision of the individuals is compromised. Blindness occurrence can lead to a harmful scenario impairing larvae to see feed and, consequently, promoting eating reduction and growing slowdown. Indeed, blindness issues and derived economical losses are increasingly attracting the interest of the aquaculture scientific community (Remø et al. 2011; Jonassen et al. 2017; Liu et al. 2023). Finally, skeletal deformities are found at a high incidence in fish hatcheries and lead to economical loss as these make fish unappealing (Eissa et al. 2021).

Regarding behaviour analysis, we found that the number of live larvae showing immobility in the culture plate was significantly higher in the stressed group (Fig. 3A). These data are in line with the results of previous studies in which exposure to ethanol increased the percentage of non-motile individuals in 7-dpf larvae (Ingebretson and Masino 2013). Presumably, these immobile larvae accumulate serious systemic alterations, promoting their paralysis. However, among larvae able to move, we found no alteration on the swimming pattern (Fig. 3D), contrary to other chronic stress-focused experiments where different patterns were reported between control and disturbed larvae (Levin et al. 2004). These results indicate that the impact of stress on behaviour could resemble a binary response.

Regarding eye development in zebrafish, similar alterations to the ones registered in the present work (Fig. 4A–D) have been previously reported in studies focused on stress evaluation (Sherpa et al. 2011; Cheng et al. 2021; Lee et al. 2023). miR-29a overexpression has been correlated to myopia (Zhu et al. 2022) and has been suggested to play a crucial role regulating ocular vasculopathy (Peng et al. 2022). Moreover, miR-29a targets some crucial mRNAs linked to RA, a potent morphogen essential for embryonic development, which participates in eye development and is critical for normal optic vesicle and anterior segment formation (Thompson et al. 2019). Thus, the deregulation in RA-signalling can result in severe ocular developmental diseases. Our qPCR results showed only a non-statistically significant trend in the expression of the studied RA receptors targeted by miR-29a: *rarga*, *rxrba* and *rxrbb* (Fig. 4E).

Regarding the registered malformations related to jaw elongation (micrognathia) and cranial skeleton development, we decided to further explore cartilage tissue in the larvae. Alcian blue staining revealed cranial skeleton to be severely affected by stress induction in the malformed larvae (Fig. 5A, B). Similar alterations in cranial encephalic cartilage development have been previously reported in zebrafish larvae exposed to different stressors, such as thermal stress (Long et al. 2012) or pollutants (Staal et al. 2018). Our analyses revealed shorter lower jaws and heads and wider M-PQ angles in the chronically stressed larvae. In ecotoxicology and drug testing experiments, the M-PQ angle has been proposed as a reliable high-throughput standard parameter to assess craniofacial outcomes after single or mixed compound exposures (Raterman et al. 2020) since this is especially affected in craniofacial malformations, such as microcephaly and micrognathia. Since collagen is a major extracellular matrix (ECM) protein forming a microfibrillar network in many tissues, including skeletal muscle, cartilage, tendon, nervous system, eye or heart, among others (Ferreira et al. 2012; Sprangers and Everts 2019; Zhao et al. 2021), we selected five miR-29a-targeted collagen genes to study gene expression in our experimental groups. miR-29a targets more than 25 collagen transcripts (Additional file 3: Table S2). The selected transcripts in the present work have been previously reported to play key roles in zebrafish larvae cranioencephalic cartilages development: *col2a1a*, *col2a1b*, *col6a2*, *col6a3* and *col11a1a* (Dale and Topczewski 2011; Tonelotto et al. 2019; Hardy et al. 2020; Reeck and Oxford 2022). Given that we found miR-29a overexpression in the stressed larvae, we expected to find a downregulation in these candidate transcripts. Globally, the results supported this hypothesis (Fig. 5E), reporting a downregulation trend in all studied collagen genes. Three of the genes showed statistically significant differences: *col2a1a*, *col6a2* and *col11a1*, corroborating the impact of exposure to successive stressors on larvae cartilage development.

## Conclusion

Our results show that the culture protocol used to induce stress in early developmental stages in zebrafish subtly affected survival percentages and did not affect hatching rates, but significantly increased malformation rates, in particular those related to cranioencephalic malformations (shorter lower jaw length, shorter head and wider M-PQ angle). Behaviour analyses demonstrated that the percentage of immobile larvae statistically significantly increased in the stressed group; interestingly, mobile larvae from both experimental groups showed similar swimming patterns. These reported physiological alterations correlated to miR-29a overexpression in stressed individuals and downregulation of essential collagen transcripts related cranioencephalic cartilage development. This study contributes to a better understanding of the effects of stressful culture conditions in early developmental stages in teleost, a crucial issue for the aquaculture industry which indicates miR-29a to be a potential molecular marker to test new larvae culture programmes in teleost species.

### Supplementary Information

Below is the link to the electronic supplementary material.

Supplementary file1 (XLSX 10 KB)

Supplementary file2 (MP4 14562 KB)

Supplementary file3 (XLSX 312 KB)

Supplementary file4 (JPG 402 KB)

## Data Availability

All data generated or analysed during this study are included in this article and its additional files.

## Abbreviations

cDNA: Complementary deoxyribonucleic acid
CNS: Central nervous system
dpf: Days post-fertilisation
ECM: Extracellular matrix
EM: Embryo medium
ER: Endoplasmic reticulum
GO: Gene ontology
HKG: Housekeeping gene
hpf: Hours post-fertilisation
H&E: Haematoxylin and eosin stain
M1: Major axis of the virtual ellipse generated from the eye area
M2: Minor axis of the virtual ellipse generated from the eye area
miRNA: Micro-ribonucleic acid
M-PQ: Meckel’s–palatoquadrate
mRNA: Messenger ribonucleic acid
NTT: Novel tank test
PBS: Phosphate-buffered saline
qPCR: Quantitative polymerase chain reaction
RA: Retinoic acid
RARs: Retinoic acid receptors
RNA: Ribonucleic acid
RNAseq: Ribonucleic acid sequencing
S^−^: Stressed samples
S^+^: Control samples
