# Supplementary figures and images for: Early stress exposure on zebrafish development: effects on survival, malformations and molecular alterations
Source: Fish Physiol Biochem. 2024 May 14;50(4):1545–62. doi: 10.1007/s10695-024-01355-0 (PMC11286684; doi:10.1007/s10695-024-01355-0)

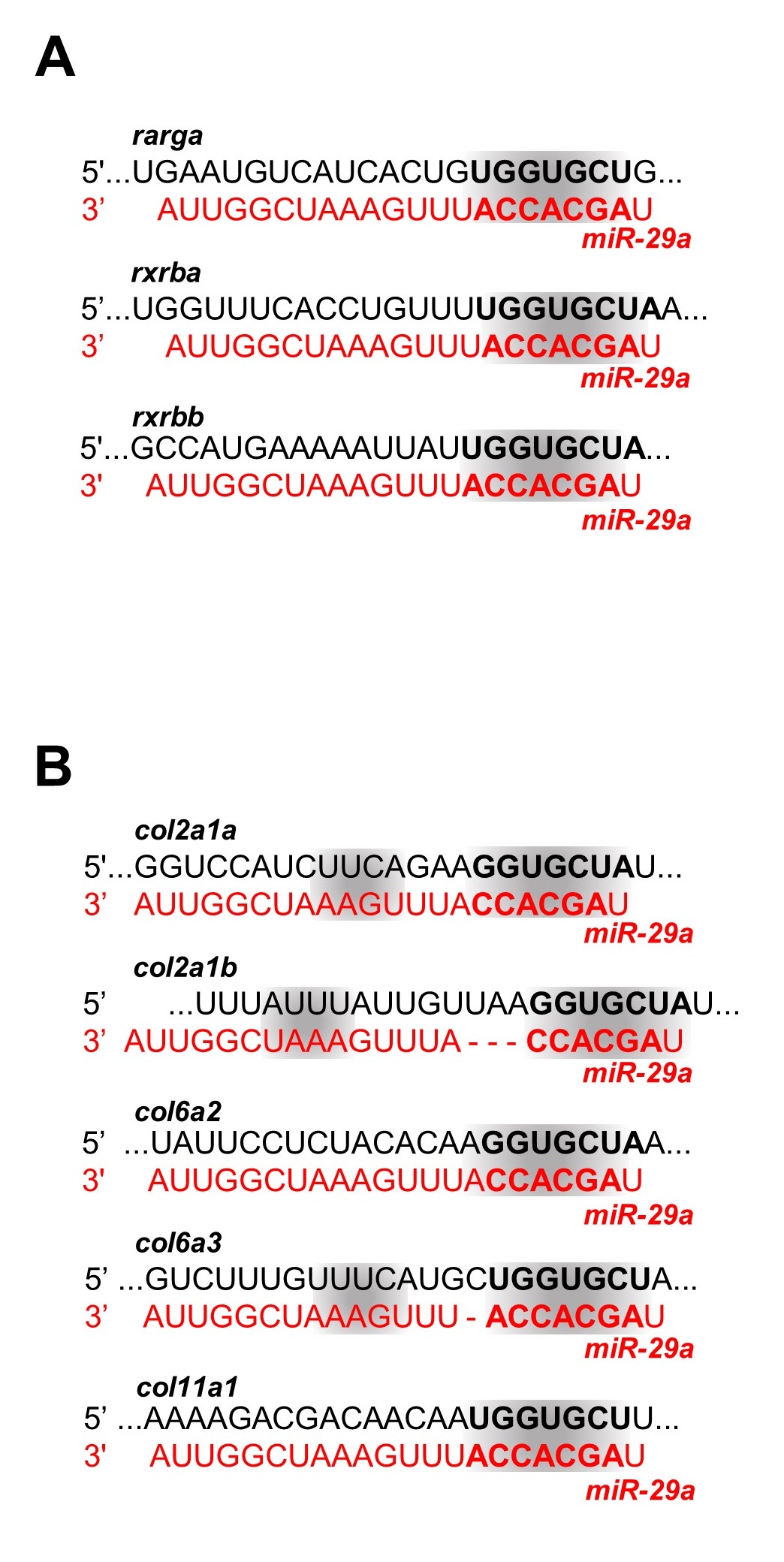

Supplement: Supplementary file 4 — Supplementary file4 (JPG 402 KB) [file 10695_2024_1355_MOESM4_ESM.jpg]
